# Multifocal epithelial hyperplasia confined to the interdental papilla of an adult Chinese man: a rare case report and literature review

**DOI:** 10.1186/s12903-023-03282-7

**Published:** 2023-09-29

**Authors:** Qianting Wang, Lehan Xu, Xiaojun Li, Mengfei Yu, Qianming Chen

**Affiliations:** Stomatology Hospital, School of Stomatology, Zhejiang University School of Medicine, Zhejiang Provincial Clinical Research Center for Oral Diseases, Key Laboratory of Oral Biomedical Research of Zhejiang Province, Cancer Center of Zhejiang University, Hangzhou, 310006 China

**Keywords:** Multifocal epithelial hyperplasia, Heck’s disease, Human papillomavirus, Gingiva, Case report

## Abstract

**Background:**

Multifocal epithelial hyperplasia (MEH), or focal epithelial hypertension (FEH), or Heck’s disease, is an uncommon, benign oral mucosal disease associated with human papillomavirus infection. It is mostly observed in indigenous populations of the world, and has been rarely reported in China. However, previous research suggested there might be a greater prevalence of MEH in the Chinese population. While predominantly involves the lips, buccal mucosa and tongue, MEH was occasionally reported to affect the hard palate and gingiva as well.

**Case presentation:**

This paper reports a case of extensive MEH lesions that confined to the interdental papilla of a Chinese male without detection of HPV, and summarizes the published gingiva-involved MEH reports from 1966 until present. The lesions were excised with an Er: YAG laser after scaling and root planning, no recurrence was observed after 6-month follow-up.

**Conclusions:**

The present report illustrates the need for clinicians to be aware of rare presentations of MEH to facilitate a prompt diagnosis and proper management. More reports are encouraged to determine a correct prevalence rate of MEH in China.

**Supplementary Information:**

The online version contains supplementary material available at 10.1186/s12903-023-03282-7.

## Background

Multifocal epithelial hyperplasia (MEH), also known as Heck’s disease, is a rare benign disease that was first described in English literature by Archard et al. (1965) [1, 2]. Previous research has suggested an association of MEH with human papillomavirus (HPV) genotypes 13 and 32 infection [3]. MEH occurrence varies from 0.02% to 35% depending on population and geographical region, it is more prevalent in indigenous populations of the Americas but is relatively rare in Asia, a recent report suggests an increased incidence in European region [2–5]. In 2013, Liu et al. reported the first two MEH cases and suggested MEH might be more prevalent in Chinese people than had been thought, as it is easy to misdiagnose [4]. Characterized by multiple, asymptomatic, soft, small raised papules or nodules in the oral cavity, predominantly on the lips, buccal mucosa, and tongue, MEH was occasionally reported to involve the hard palate and gingiva as well [2, 3, 6]. A recent systematic review shortlisted 95 published cases from 1966 to 2020, and found 8 out of 95 MEH cases reported gingiva lesions [2]. This case aims to summarize the published gingival-involved MEH cases and encourage more awareness of MEH in the Chinese population.

## Case presentation

A 37-year-old married male with a history of smoking 3-5 beedis/day for the 20 years was referred to the Stomatology Hospital, Zhejiang University School of Medicine, on November 30^th^, 2020. His chief complaint was the isolated slow-growing exophytic growth on the anterior gums for over 10 years, and he reported lightening of the lesions after supragingival scaling the previous week. The patient denied significant medical or family history, although he mentioned a history of ablative therapy for a single, non-recurring genital wart. Unfortunately, relevant medical records or pathological evidence were not available to confirm the diagnosis.

**Fig. 1:**
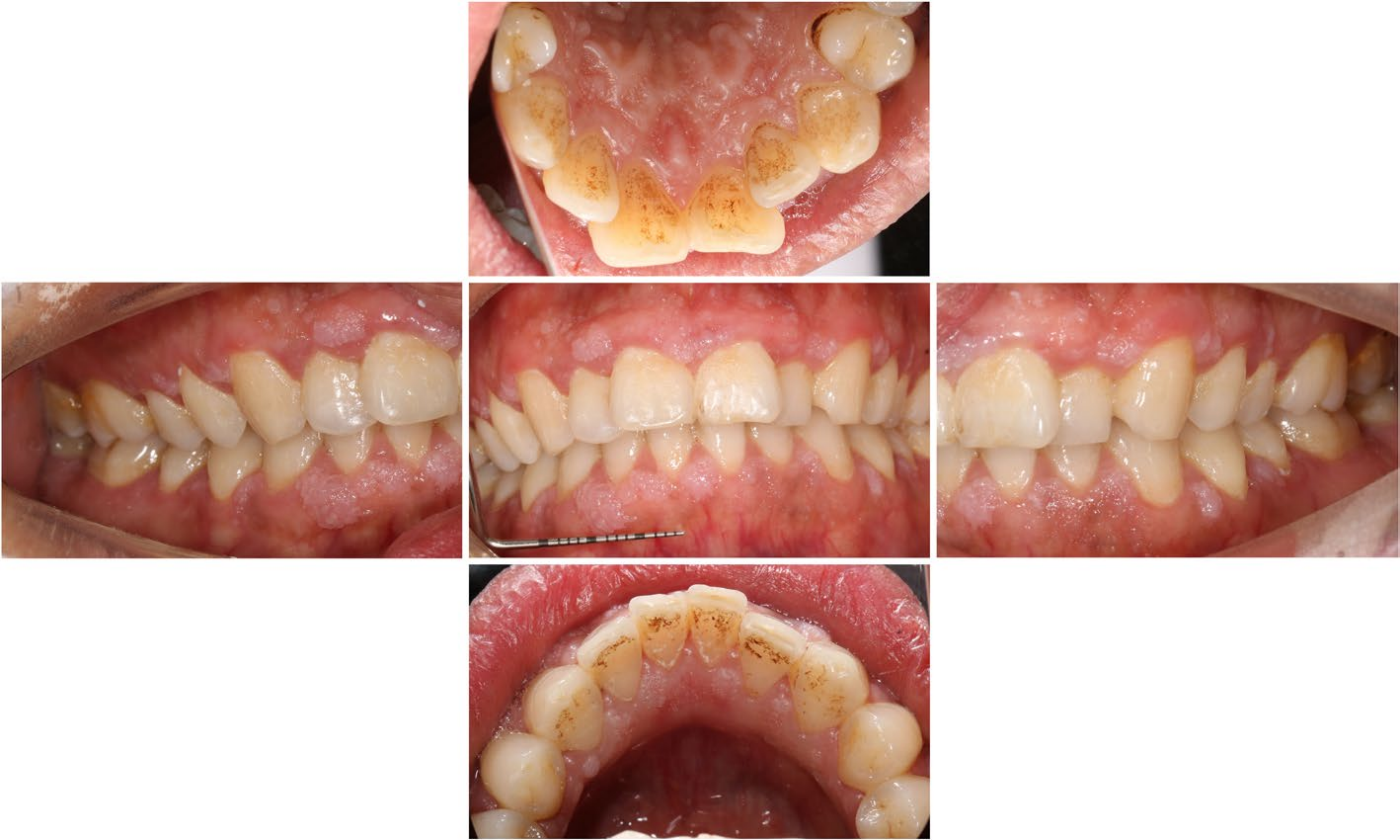
Intraoral clinical examination showed multiple diffuse white papules with varying sizes on the interdental gingiva

Clinical examination revealed multiple demarcated, exophytic, pedunculated, soft, pale papules on the labial and lingual interdental papilla areas of the anterior and molar teeth. The lesions ranged in size from 1 to 5 mm and were coalescent. There was also significant accumulation of plaque and subgingival calculus (Fig. 1). Extraoral examination showed no abnormalities and none of his family had similar lesions. Laboratory test results were unexceptional including normal complete blood counts and coagulation profile, negative serology for human immunodeficiency virus (HIV), syphilis, hepatitis B virus (HBV), and hepatitis C virus (HCV).

The provisional diagnosis was multifocal squamous papilloma, with differential diagnoses of condyloma acuminatum, MEH and verruciform xanthoma. Two large lesions in the right posterior buccal gingiva were excised, specimen was processed for histopathological examination and polymerase chain reaction (PCR) analysis using consensus primers GP5 + /6 + and MY09/11, and type-specific primers for HPV16, 18, 13, and 32 (details of methods are submitted in Supplementary materials). Cells from the lesions were collected for the PCR-reverse dot blot (RDB) HPV (6、11、16、18、26、31、33、35、39、40、42、43、44、45、51、52、53、56、58、59、66、68、73、81、83) genotyping assay.

**Fig. 2:**
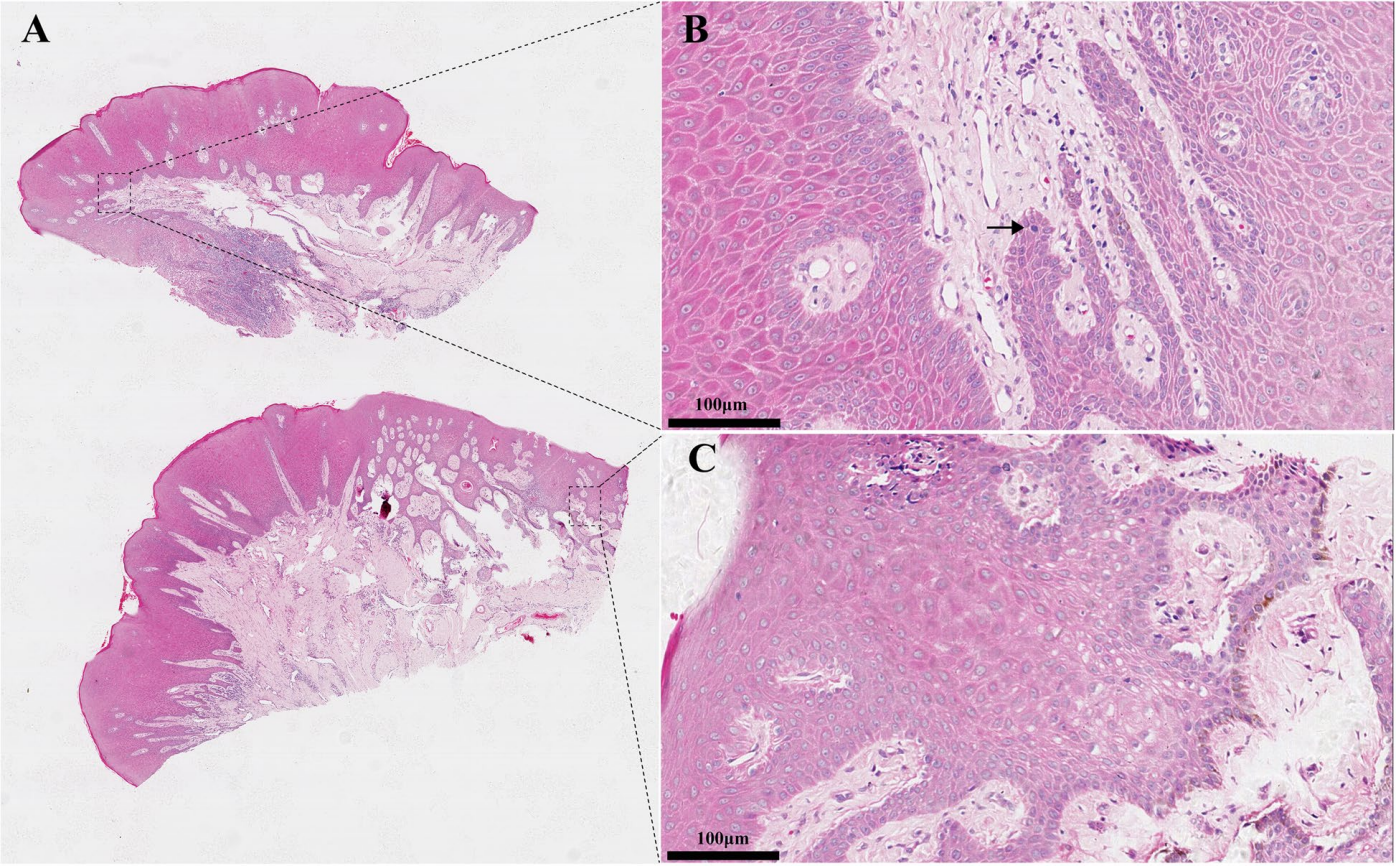
**A** Photomicrographs of the lesion showing epithelial hyperplasia with acanthosis and parakeratosis, and elongated, thickened, and anastomosed rete ridges, with fibrovascular connective tissue cores (hematoxylin and eosin HE, original magnifications 40 ×). **B** A mitosoid body in the stratum spinosum (HE, 400 × , arrow). **C** koilocytes with a perinuclear halo in the upper layers of the epithelium (HE, 400 ×)

Histological analysis showed hyperplastic squamous epithelium with papillomatosis, acanthosis, hyperkeratosis, and parakeratosis. Elongated rete ridges anastomosed horizontally and koilocytosis were noted within the upper layers of the epithelium with a perinuclear halo, with rare mitosoid bodies (Fig. 2). The tissue was negative for HPV DNA on RDB genotyping and PCR with universal primers. Although HPV 13 and 16 were detected by PCR subtyping (Supplementary materials, Fig. S1), subsequent Sanger sequencing of the purified PCR products and BLAST analysis (http://blast.ncbi.nlm.nih.gov) revealed the sequences were human genes and so no HPV was detected in this case.

Clinical and histopathological features were consistent with a diagnosis of MEH. Scaling and root planning (SRP) was prescribed along with oral hygiene instructions [7]. Topical application of imiquimod was also prescribed, however, the patient was not willing to quit smoking or comply with the use of imiquimod due to inconvenience and discomfort [6]. No recurrence of the previously excised lesions in the right posterior buccal gingiva or changes of the remaining lesions were seen at 3-month and 11-month follow-up (Supplementary materials Figs. S2, S3). The remaining papules were excised with an Er: YAG laser, the excision sites healed without complication and there was no recurrence over a period of 6 month (Supplementary materials Figs. S4, S5, S6) [8, 9].

## Discussion and conclusions

MEH is a rare benign proliferative disease of the oral mucosa associated with HPV infection. Two clinical forms have been described in the literature: papulonodular and papillomatous. The papulonodular variant occurred mainly on the lining mucosa and is more common than the papillomatous type, which occurred on the masticatory mucosae such as the attached gingiva and the tongue [3, 7]. MEH is found predominantly in children and adolescents in certain populations, a variable female predilection and familial distribution have been reported [2, 3, 6]. We present a case of MEH on the interdental gingiva in a 37‐year‐old Chinese male with no family history of the disease.

The MEH lesions tend to occur on the buccal and labial mucosa, it rarely affects the gingiva or the hard palate. Sethi et al. shortlisted 95 published MEH cases from 1966 to 2020, and found 8 out of 95 total cases reported gingiva lesions [2]. The latest systematic review conducted by Di Spirito F, et al. categorized 7 gingiva-involved MEH cases [10]. Seventeen reports of gingiva-involved MEH are tabulated in Table 1. The first gingiva-involved MEH case was reported by Van der Waal et al. in 1975 [11]. Multiple, symptomless, papillomatous-like swellings was found on the lower and upper lips, buccal mucosa, gingiva and the borders of the tongue of a 12-year-old black boy, the hard palate was involved as well. Two years later, Starink TM et al. reported two MEH cases involving gingiva of a 9-year-old girl and a 4-year-old boy, respectively [12]. In 1982, Lutzner M et al. described MEH lesions on the gingiva of a 10-year-old boy [13]. In 1993, Morrow et al. reported the first gingiva-involved MEH case in adult: MEH lesions on the lips and gingiva of a 23-year-old black female; the report included no intraoral pictures [14]. In 2003, Akyol et al. reported MEH involving the lips, buccal mucosa, gingiva, and tongue of a 17‐year‐old male [8]. In the same year, Nartey et al. reported six MEH cases, two of which involved gingiva, regression disparity between the papulonodular and the papillomatous variants was observed in this report [7]. In another case, HPV 13-related MEH lesions were found on the gingival mucosa of a 14-year-old American Indian girl [15]. HPV 13 infection was also confirmed in the gingiva-involved MEH case reported by Bennett et al. [16]. In 2013, Prabhat et al. reported extensive coalescent papillary projections involving the maxillary and mandibular gingiva, palate, tongue, buccal mucosa along with skin lesions in a 65-year-old Indian female, with HPV 16 DNA detected [17]. In 2014, Park et al. described multiple pinkish gingival nodules near the attached gingiva of a male related to prosthesis without detection of any HPV subtype [18]. Agnew C et al. reported a 5-year-old girl presented with papulonodular lesions on the labial mucosa, along with papillomatous nodules on the gingiva and tongue in 2017 [19]. The latest two gingiva-involved MEH cases were reported in 2023: Turco M et al. described soft pedunculated papules located on the buccal mucosa, hard palate and oral commissures of a 12‐year‐old Peruvian girl, and revealed the presence of HPV-32 [20]. Santana-Gutierrez A et al. present successful treatment of MEH on gingival, buccal mucosa and lips of a 14-year-old girl with intralesional immunotherapy [21].

**Table 1:** Summary of the characteristics of related cases

Authors, year	Age, gender	Sites	Intraoral clinical features	Associated HPV subtypes and methods	Treatment and outcome
Van der Waal et al. (1975) [11]	12, M	Lips, buccal mucosa, gingiva, tongue and palate	Multiple, symptomless, papillomatous-like swellings	Not mentioned	No treatment, lesions hardly changed during 6-month follow-up
Starink TM et al. (1977) [12]	9, F	Lips, commissures, buccal mucosa, gingiva, palate and anterior faucial pillars	soft papules, pink or whitish with a flat to slightly verrucous surface, 1-10 mm in diameter	Not mentioned	Treated with 0.05% vitamin A acid in Orabase. The lesions were unchanged 6 months later
Starink TM et al. (1977) [12]	4, M	Lips, commissures, buccal mucosa, tongue, palate, gingiva and the floor of mouth	20 papules, smaller than 4 mm in diameter	Not mentioned	No treatment. Unknown (the patient failed to return for re-examination)
Lutzner M et al. (1982) [13]	10, M	labial and buccal mucosa, gingiva, tongue, dorsa of the hands and forehead	multiple, soft, slightly elevated, oval to round papules, 2-5 mm in diameter, with a pink or white surface	Not mentioned	Treated with shaving and electrocoagulation
Morrow DJ et al. (1993) [14]	23, F	Lips, maxillary and mandibular gingiva, and palate	Multiple soft, elevated, sessile, isolated, non-ulcerated lesions, with whitish surfaces, 2-3 mm in diameter	No HPV detected. Immunoperoxidase staining and microscopy	Not mentioned
Akyol A et al. (2003) [8]	17, M	Lip, buccal mucosa, gingiva, and tongue	Numerous papillomatous and verrucous lesions with a tendency to coalesce	Not mentioned	CO2 laser therapy combined with interferon alpha‐2b, no recurrence within 2-year follow-up
Nartey NO et al. (2003) [7]	4, F	Buccal and labial mucosa, floor of mouth, tongue and gingiva	Multiple whitish papillomatous and pink papulonodular lesions with sessile bases	Not mentioned	No treatment, lesions present 3 years after initial visit
Nartey NO et al. (2003) [7]	12, F	Gingiva	Multiple papillomatous lesions	Not mentioned	No treatment, lesions present 3 and half years after initial visit
Martins WD et al. (2006) [15]	14, F	Mandibular left buccal gingiva	4 elevated, sessile, smooth-surfaced, asymptomatic nodules, 1-3 mm in diameter	HPV 13. PCR	Surgical excision, no recurrence within 2-year follow-up
Bennett LK et al. (2009) [16]	9, F	Buccal, gingival, and labial mucosa	Multiple soft, pink, noninflammatory exophytic, flat-topped 2 to 6 mm papules	HPV 13. In situ hybridization and PCR	No treatment
Prabhat MP et al. (2013) [17]	65, F	Maxillary and mandibular gingiva, palate, tongue, buccal mucosa, and skin	Extensive coalescent papillary lesions	HPV 16. PCR	No treatment (the patient denied laser ablation)
Liu N et al. (2013) [4]	33, F	Mandibular anterior labial gingiva	A number of pale, protruded, soft, asymptomatic papules, 1-3 mm in diameter	HPV 32. PCR	No treatment
Park MW et al. (2014) [18]	53, M	Right upper posterior buccal gingiva and left lower lingual gingiva	Multiple small pebbly and white or slightly reddish nodules	No HPV DNA detected. HPV DNA chip assay	Surgical excision. No recurrence at18-month follow-up
Agnew C et al. (2017) [19]	5, F	Labial mucosa, attached gingiva and lateral borders of the tongue	Labial mucosa: multiple sessile papulonodular lesions coalesced, forming a cobblestoned, fissured appearance, 0.5-5.0 mm in diameter. Gingiva and tongue: pale pink, pebbly papillomatous nodules	Not mentioned	No treatment. All lesions spontaneously resolved after 15 months
Betz. SJ et al. (2019) [5]	A clinical image of generalized MEH lesions across the gingival of both arches was illustrated in the review without detailed information				
Turco M et al. (2023) [20]	12, F	buccal mucosa, hard palate, and oral commissures	multiple exophytic papules of different sizes, having the same color as the surrounding epithelium	HPV 32. PCR	Not mentioned
Santana-Gutierrez A et al. (2023) [21]	14, F	gingival, buccal mucosa, lips and tongue	multiple mucosal-colored, soft papules	Not mentioned	Intralesional immunotherapy with MMR vaccine, combined with the application of TCA. Lesions resolved after five sessions with no adverse reactions

*M* Male, *F* Female, *HPV* Human papillomavirus, *PCR* Polymerase chain reaction, *MMR* Measles, mumps and rubella, *TCA* Trichloroacetic acid

There is little documentation on MEH in Chinese population. Two brief MEH case reports were retrieved in Chinese literature database: Chen et al. reported MEH on the buccal mucosa, labial mucosa, palate and attached gingiva of a 31-year-old male in 1986, attached with histopathological pictures [22]. In 2006, Zhang et al. reported a single MEH lesion on the gingiva of a 26-year-old male [23]. Unfortunately, neither of the two reports included intraoral pictures. These two cases were not included in Table 1. In 2013, Liu et al. reported the first two MEH cases in Chinese population in English literature, one of which described several 1–3-mm diameter papules on the mandibular anterior labial gingiva in a woman [4]. In the present case, the man had widespread large lesions across the interdental papilla of both arches. The lesions of MEH almost always arise on multiple sites in each individual, single-site involvement by multiple lesions is very uncommon [3]. Apart from an illustration of similar lesions in a recent review, this is the fifth report of MEH confined to the gingiva [3–5, 7].

MEH is strongly associated with HPV infection. Whilst HPV 13 and 32 are the most frequently detected subtypes, co-infection or infection with other HPV-types like HPV 6, 11, 16, 18, 31, 39, 40, 51, 52, 55, 58, 66, 68, 69, 71, 74 and 90 are also occasionally described [3, 6, 17, 24–26]. Currently, HPV 13, 16 and 32 have been detected in previous gingiva-involved MEH cases. Although no case of malignant transformation in MEH has been described, it is currently unknown what effect MEH lesions coinfected with high-risk genotypes relating to malignant changes of the oral cavity (16 and 18) have on a possible malignant transformation [6]. Considering the self-reported history of suspicious genital wart ablation that suggests possible oral HPV infection through sexual behavior or self-inoculation, we conducted type-specific PCR to detect HPV 13, 16, 18 and 32. However, no HPV DNA was detected in this case. PCR analysis is the most commonly used tool for the detection of HPV DNA. In this case, the sequences of HPV 13 and 16 detected by type-specific PCR were further excluded by Sanger sequencing and BLAST analysis, indicating the requirement of DNA sequencing to prevent false positives findings.

Along with HPV etiology, low socioeconomic status, crowded living conditions, malnutrition, tobacco exposure, immunosuppression, and poor hygiene are also associated with MEH [3, 6]. A previous case reported spontaneous regression of the buccal and labial mucosa in MEH after 4 months following enhanced oral hygiene, suggesting a potential association between MEH and oral hygiene [27]. The possible causes of MEH in this patient would include poor oral hygiene and smoking, therefor SRP and tobacco control were advised to the patient. However, no regression was observed in this case after SRP. This may be due to the different distribution areas between the two cases. In this case, lesions were located on the attached gingiva where the underlying connective tissue was dense, in contrast to the loose underlying dermis of the buccal and labial mucosa [7]. Moreover, smoking habits and old age may have also contributed to the persistence of the lesions. Indeed, spontaneous regression of the gingival MEH lesions had only been observed on a 5-year-old girl [19]. The exact roles of factors such as poor oral hygiene and tobacco in subsets of MEH patients remain unclear and merit further studies.

Considered as a benign disease, MEH does not require treatment unless the lesions cause functional or cosmetic concerns [3]. In this case, the patient opted to undergo SRP and surgical excision of the more conspicuous lesions in the buccal gingiva. To our knowledge, this is the first report including SRP as part of the treatment plan. Although no remission of the lesions was seen after 11 months, the lesions also did not progress or recur, indicating a stable disease status and so the remaining lesions were excised using an Er: YAG laser. No recurrence was observed after 6 months, indicating a favorable long-term prognosis of the condition. The patient was suggested to visit us on a regular basis to monitor the gingival lesions.

Gingiva-involved MEH has been uncommonly reported, particularly when it has widespread lesions confined to the gingiva [2]. This may be due to the low incidence of MEH on the gingiva or misdiagnosis of the disease. In this case, the patient was initially misdiagnosed with extensive epulis before referral to our department. Clinicians need to be aware of such rare presentations of MEH to facilitate a prompt diagnosis and proper management. Back in 2013, Liu et al. has suggested MEH may be more prevalent in Chinese people than has been thought, as it is easy to misdiagnose [4]. In addition, Di Spirito F, et al. reported that the cases of MEH waited an average of one and a half years after the appearance of the lesions before seeing a specialist because the lesions are often asymptomatic [10]. In the present case, the appearance of the MEH lesions occurred almost ten years before the patient sought medical care. Consequently, it is reasonable to assume that lacking awareness and concern about the lesions may lead to a low consultation rate, which attributes to the lower prevalence rate of MEH in the Asian population. Therefor more reports are required to determine a correct prevalence rate of MEH in China.

MEH is reported to be the most frequently diagnosed HPV-related oral lesions in healthy pediatric patients [10]. Although no HPV DNA was detected in this case, and there is no evidence that MEH has malignant potential, the authors propose it is necessary to raise awareness of MEH among population and clinicians. MEH has recently been introduced into the latest version of the Chinese oral histopathology textbook published in 2020. We believe this case report will prompt the Chinese clinicians to pay more attention to MEH.

## Conclusions

This case highlights a rare presentation of MEH confined to the interdental gingiva in a Chinese male. More awareness about MEH is encouraged in population and in clinicians, especially the Chinese clinicians. Future research is required to gain further understanding of the pathogenesis of MEH, its association with viral infection, oral hygiene, age and smoking.

### Supplementary Information

**Additional file 1.**

## Data Availability

The datasets generated during the current study are available from the corresponding author on reasonable request.

## Abbreviations

MEH: Multifocal epithelial hyperplasia
HPV: Human papillomavirus
HIV: Human immunodeficiency virus
HBV: Hepatitis B virus
HCV: Hepatitis C virus
PCR: Polymerase chain reaction
RDB: Reverse dot blot
SRP: Scaling and root planning

## References

[1] Archard HO, Heck JW, Stanley HR (1965). Focal epithelial hyperplasia: an unusual oral mucosal lesion found in Indian children. Oral Surg Oral Med Oral Pathol.

[2] Sethi S, Ali A, Ju X, Antonsson A, Logan R, Jamieson L (2022). An update on Heck's disease-a systematic review. J Public Health (Oxf)..

[3] Said AK, Leao JC, Fedele S, Porter SR (2013). Focal epithelial hyperplasia - an update. J Oral Pathol Med.

[4] Liu N, Li Y, Zhou Y, Zeng X (2012). Focal epithelial hyperplasia (Heck's disease) in two Chinese females. Int J Oral Maxillofac Surg.

[5] Betz SJ (2019). HPV-related papillary lesions of the oral mucosa: a review. Head Neck Pathol.

[6] Bendtsen SK, Jakobsen KK, Carlander AF, Grønhøj C, von Buchwald C (2021). Focal Epithelial Hyperplasia. Viruses.

[7] Nartey NO, Newman MA, Nyako EA (2002). Focal epithelial hyperplasia: report of six cases from Ghana. West Africa J Clin Pediatr Dent.

[8] Akyol A, Anadolu R, Anadolu Y, Ekmekci P, Gürgey E, Akay N (2003). Multifocal papillomavirus epithelial hyperplasia: successful treatment with CO2 laser therapy combined with interferon alpha-2b. Int J Dermatol.

[9] Nguyen JT, Allen CT, Dodge JT, Van Doorslaer K, McBride AA, Pavletic SZ, Mays JW (2021). HPV32-related Heck's disease in a chronic graft-versus-host disease patient with long-term successful KTP laser treatment: a rare case report. Clin Case Rep.

[10] Di Spirito F, Pantaleo G, Di Palo MP, Amato A, Raimondo A, Amato M (2023). Oral Human Papillomavirus Benign Lesions and HPV-Related Cancer in Healthy Children: A Systematic Review. Cancers (Basel).

[11] van der Waal I, ten Bruggenkate CM, van der Kwast WA (1975). Focal epithelial hyperplasia in a child from Surinam. Int J Oral Surg.

[12] Starink TM, Woerdeman MJ (1977). Focal epithelial hyperplasia of the oral mucosa. Report of two cases from the Netherlands and review of the literature. Br J Dermatol.

[13] Lutzner M, Kuffer R, Blanchet-Bardon C, Croissant O (1982). Different papillomaviruses as the causes of oral warts. Arch Dermatol.

[14] Morrow DJ, Sandhu HS, Daley TD (1993). Focal epithelial hyperplasia (Heck's disease) with generalized lesions of the gingiva. A case report J Periodontol.

[15] Martins WD, de Lima AA, Vieira S (2006). Focal epithelial hyperplasia (Heck's disease): report of a case in a girl of Brazilian Indian descent. Int J Paediatr Dent.

[16] Bennett LK, Hinshaw M (2009). Heck's disease: diagnosis and susceptibility. Pediatr Dermatol.

[17] Prabhat MP, Raja Lakshmi C, Sai Madhavi N, Bhavana SM, Sarat G, Ramamohan K (2013). Multifocal epithelial hyperplasia of oral cavity expressing HPV 16 gene: a rare entity. Case Rep Dent.

[18] Park MW, Cho YA, Kim SM, Myoung H, Lee JH, Lee SK (2014). Focal epithelial hyperplasia arising after delivery of metal-ceramic fixed dental prosthesis. J Adv Prosthodont.

[19] Agnew C, Alexander S, Prabhu N (2017). Multifocal epithelial hyperplasia. J Dent Child (Chic).

[20] Turco M, Magnaterra E, Bisanzi S, Cannistrà S, Paganini I, Sani C, Pisano L (2023). Heck's disease: a diagnostic challenge. Int J Dermatol.

[21] Santana-Gutierrez A, Pérez-Garza DM, Ocampo-Candiani J, Alba-Rojas E (2023). Intralesional immunotherapy with MMR vaccine in a paediatric case of focal epithelial hyperplasia. Australas J Dermatol..

[22] Chen J, Yang L (1986). Focal epithelial hyperplasia (with histopathological report) (in Chinese). J Pract Stomatol.

[23] Zhang P, Liu H (2007). Focal epithelial hyperplasia on gingiva: a case report (in Chinese). J Modern Stomatol.

[24] Brehm MA, Gordon K, Firan M, Rady P, Agim N (2016). Case report of focal epithelial hyperplasia (Heck's Disease) with Polymerase chain reaction detection of human papillomavirus 13. Pediatr Dermatol.

[25] Jiménez Aguilar SM, Rodríguez DL, Muñoz Estrada VF, Cázarez Salazar SG, Velarde Félix JS, Méndez Martínez RS (2023). Great diversity of oncogenic human papillomaviruses is revealed in an outbreak of multifocal epithelial hyperplasia. J Am Acad Dermatol..

[26] Khanal S, Cole ET, Joh J, Ghim SJ, Jenson AB, Rai SN, Trainor PJ, Shumway BS (2015). Human papillomavirus detection in histologic samples of multifocal epithelial hyperplasia: a novel demographic presentation. Oral Surg Oral Med Oral Pathol Oral Radiol.

[27] Ghalayani P, Tavakoli P, Eftekhari M, Haghighi MA (2015). Oral focal epithelial hyperplasia: report of three cases. Turk Patoloji Derg.

