# Supplementary material for: Multifocal epithelial hyperplasia confined to the interdental papilla of an adult Chinese man: a rare case report and literature review
Source: BMC Oral Health. 2023 Sep 29;23:699. doi: 10.1186/s12903-023-03282-7 (PMC10540325; doi:10.1186/s12903-023-03282-7)

**SUPPLYMENTARY MATERIAL**

**Multifocal epithelial hyperplasia confined to the interdental papilla of an adult Chinese man: a rare case report and literature review**

**Materials and methods**

**PCR-reverse dot blot (RDB) assay**

RDB HPV genotyping assay was conducted using a commercial Kit (Promotor, Acon labs Inc., Hangzhou, China), according to the manufacturer’s instructions.

**DNA extraction, amplification and sequencing**

Specimen DNA was extracted using Tiangen DNA tissue extraction kit (Tiangen Bio., Beijing, China) according to the manufacturer’s instructions. The DNA samples were stored at 20℃. DNA integrity were tested by amplification of a 110 bp segment of human [β](https://baike.baidu.com/item/%CE%B2%E7%B3%BB%E6%95%B0/6685182)-globin gene (Table S1). Amplification reactions were performed using Premix Taq^TM^ version 2.0 plus dye kit (Takara Bio., Japan), with a negative control (sterile water in place of DNA). HPV universal primers GP5+/6+ and MY09/11, and subtype specific primers were listed in Table S1. The PCR products were analyzed via 3% agarose gel electrophoresis and ethidium bromide staining. Purified PCR products were sequenced on an ABI 3730XL sequencer by Tsingke Biotechnology Co., Ltd, China., and then compared with the sequences of HPV 13 and 16 using Blast analysis (http://blast.ncbi.nlm.nih.gov).

*Table S1.* Oligonucleotides used for PCR amplification and detection.

| Oligonucleotides | Sequences | Products |
| --- | --- | --- |
| [β](https://baike.baidu.com/item/%CE%B2%E7%B3%BB%E6%95%B0/6685182)-globin | 5’-ACACAACTGTGTTCACTAGC-3’  5’-CAACTTCATCCACGTTCACC-3’ | 110bp |
| GP5+/GP6+ | 5’-TTTGTTACTGTGGTAGATACTAC-3’  5’-GAAAAATAAACTGTAAATCATATTC-3’ | 150bp |
| MY09/11 | 5’-CGTCCMARRGGAWACTGATC-3’  5’-GCMCAGGGWCATAAYAATGG-3’ | 450bp |
| HPV13 | 5’-TATAGTGTGGCGAGGACAGTTTC-3’  5’-TAGCACATCCAAAATTGACTGC-3’ | 147bp |
| HPV32 | 5’-GTAACCGGACAATGGGAGGTA-3’  5’-GGTGTTGTATAGGTCGGTGGTT-3’ | 140bp |
| HPV16 | 5’-TCAAAAGCCACTGTGTCCTG-3’  5’-CGTGTTCTTGATGATCTGCA-3’ | 120bp |
| HPV18 | 5’-ACCTTAATGAAAAACGACGA-3’  5’-CGTCGTTGGAGTCGTTCCTG-3’ | 100bp |

*Figure S1.* Representative agarose gel electrophoresis of HPV subtype-specific PCR reaction.


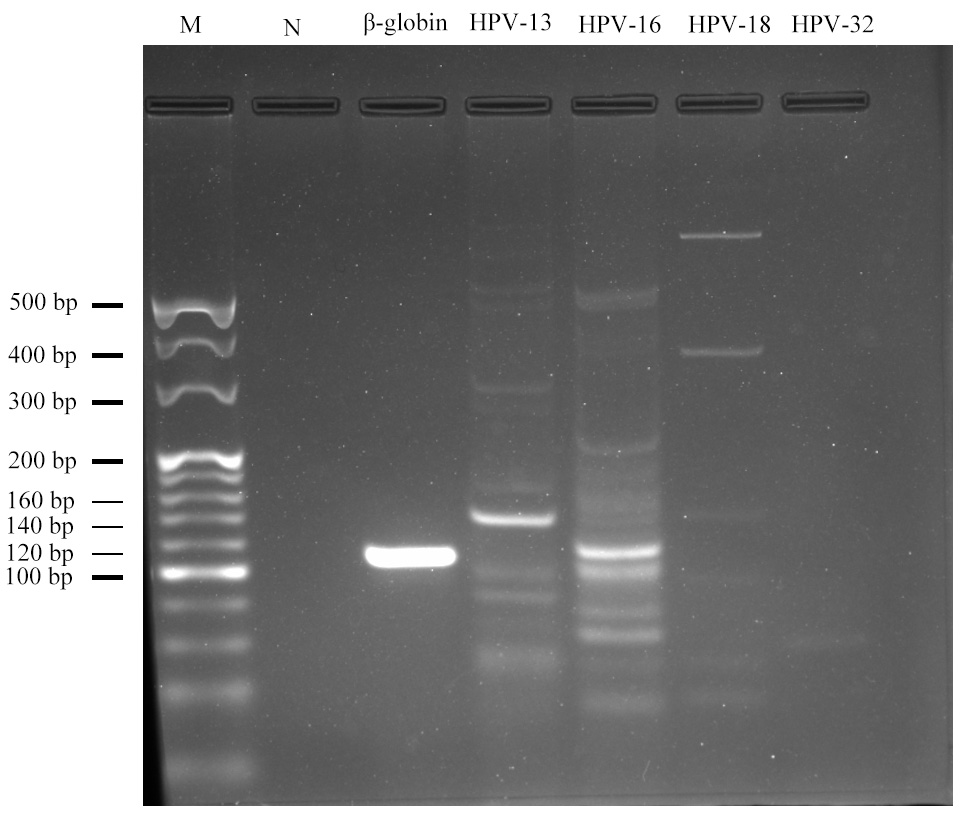


*Figure S1.* Representative agarose gel electrophoresis of HPV subtype-specific PCR. PCR primers used: [β](https://baike.baidu.com/item/%CE%B2%E7%B3%BB%E6%95%B0/6685182)-globin, lane 3; HPV subtype 13, lane 4; HPV subtype 16, lane 5; HPV subtype 18, lane 6; HPV subtype 32, lane 7; M is marker; N is water (template negative), control.

*Fig. S2* 3-month revisit periodontal maintenance after excision and scaling and root planning (SRP).


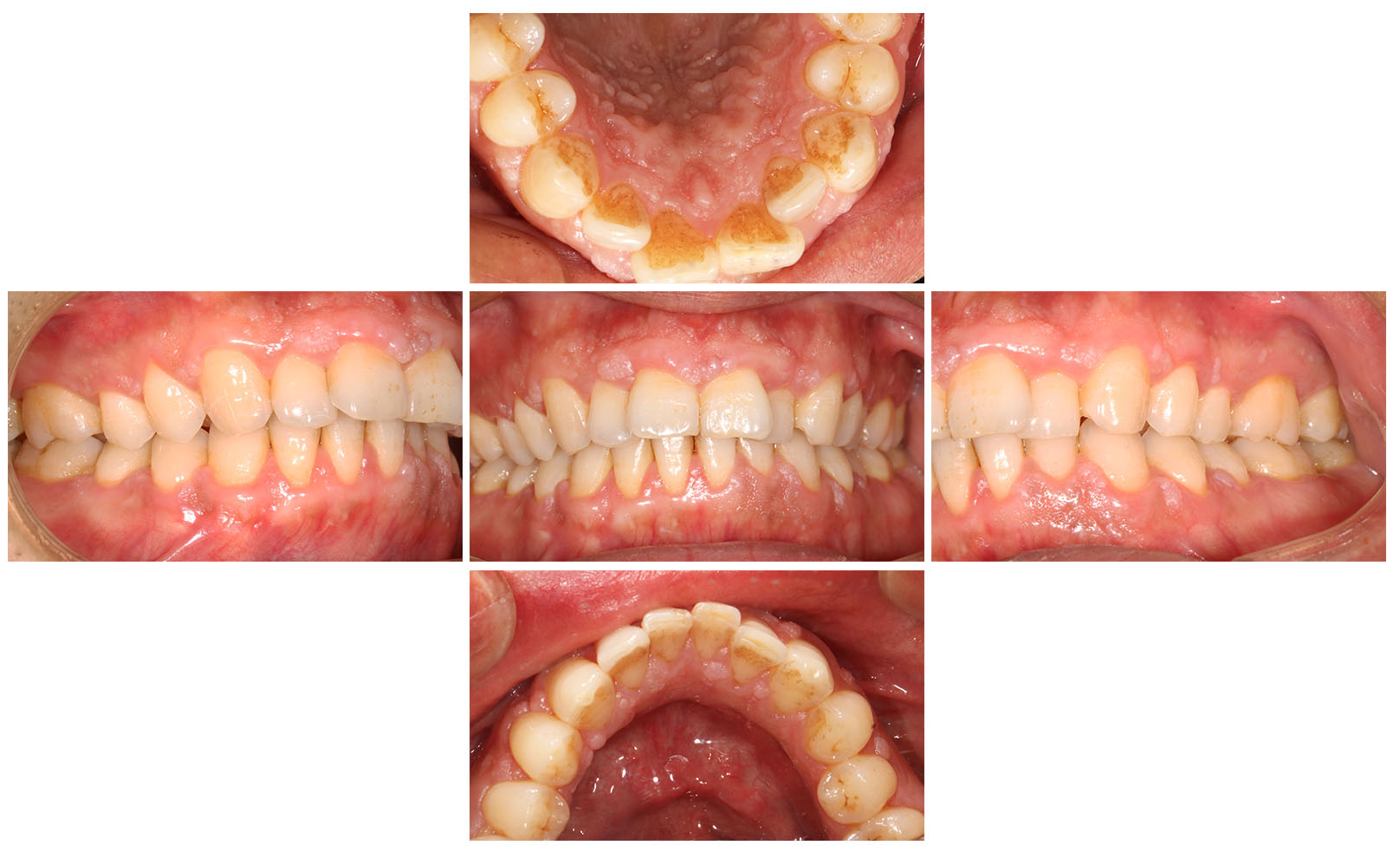


*Fig. S3* Clinical appearance at the 11-month follow up, no recurrence or changes of the remaining lesions were observed.


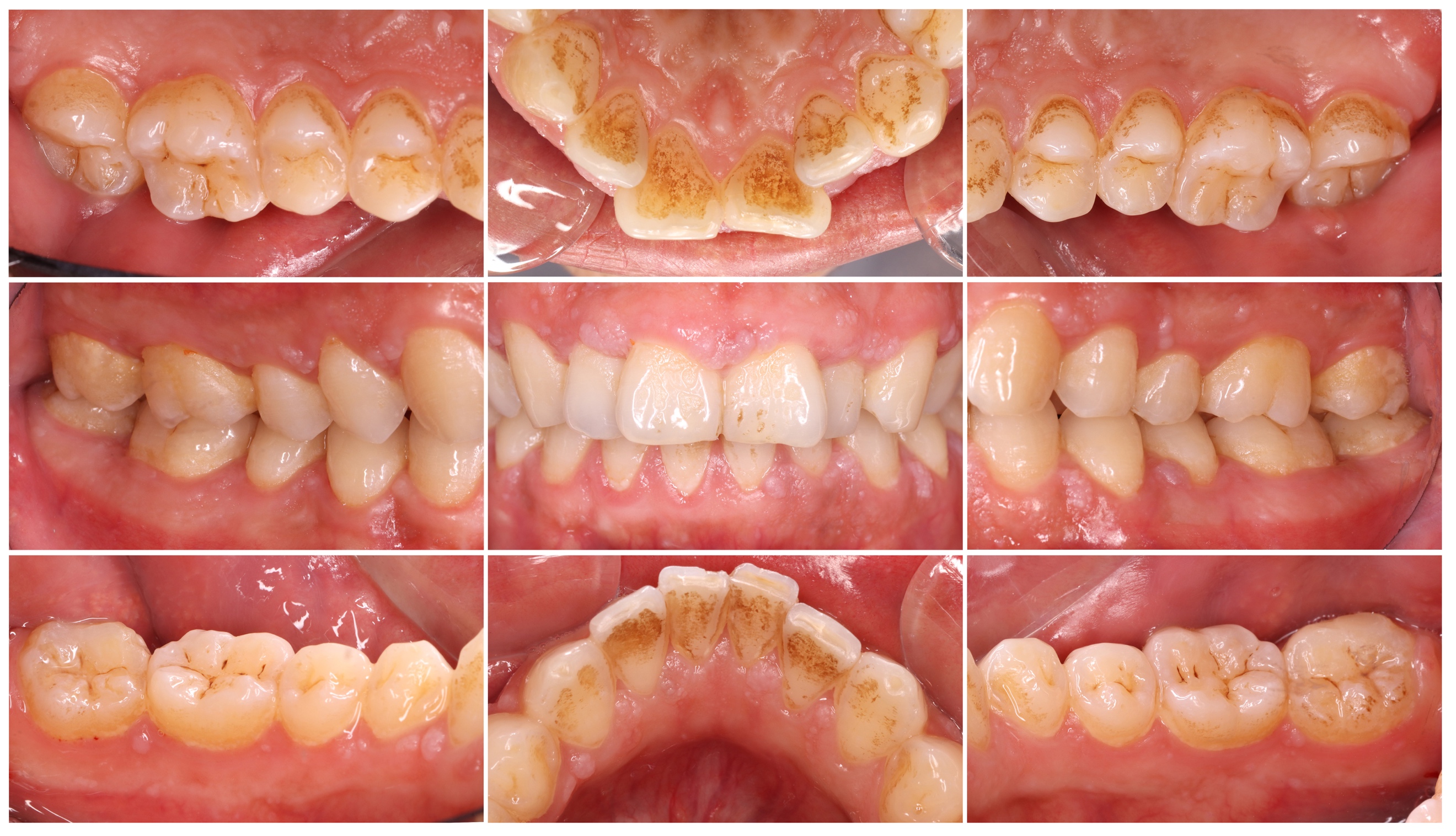


*Fig. S4* Clinical appearance of the lesions after gingivectomy with Er: YAG laser.


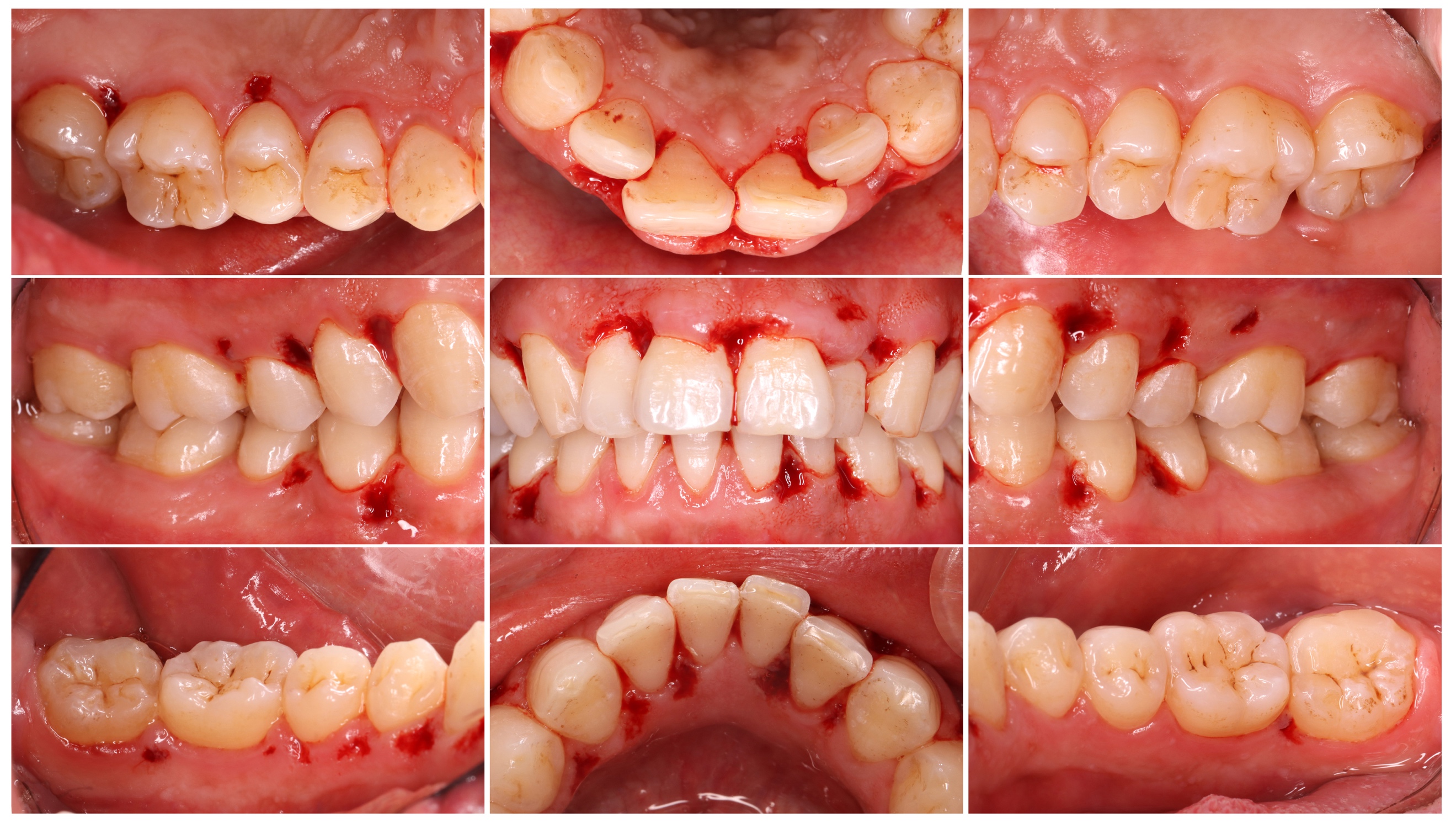


*Fig. S5* 1 month visit after Er: YAG laser excision.


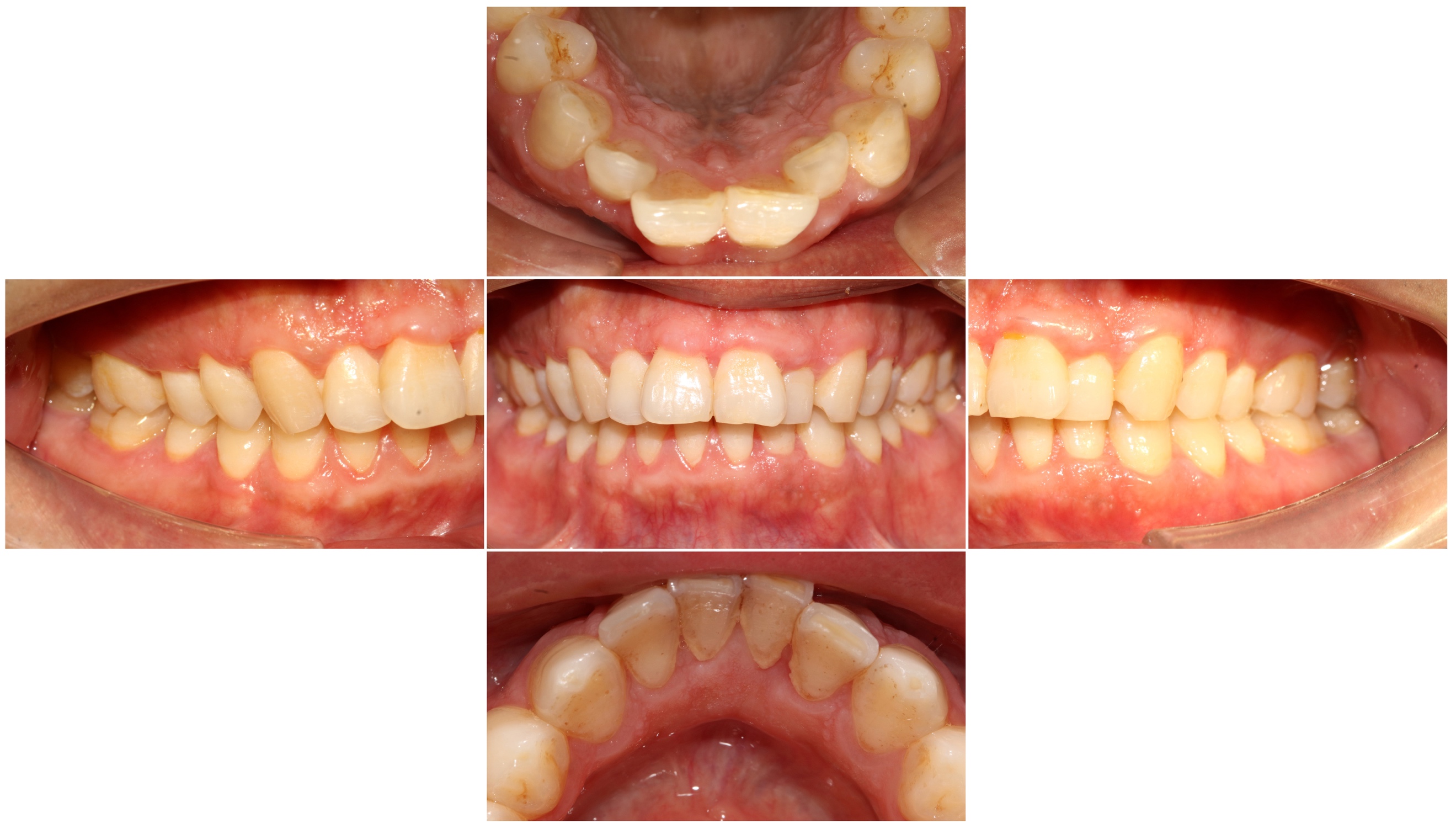


*Fig. S6* 6 month visit after Er: YAG laser excision.


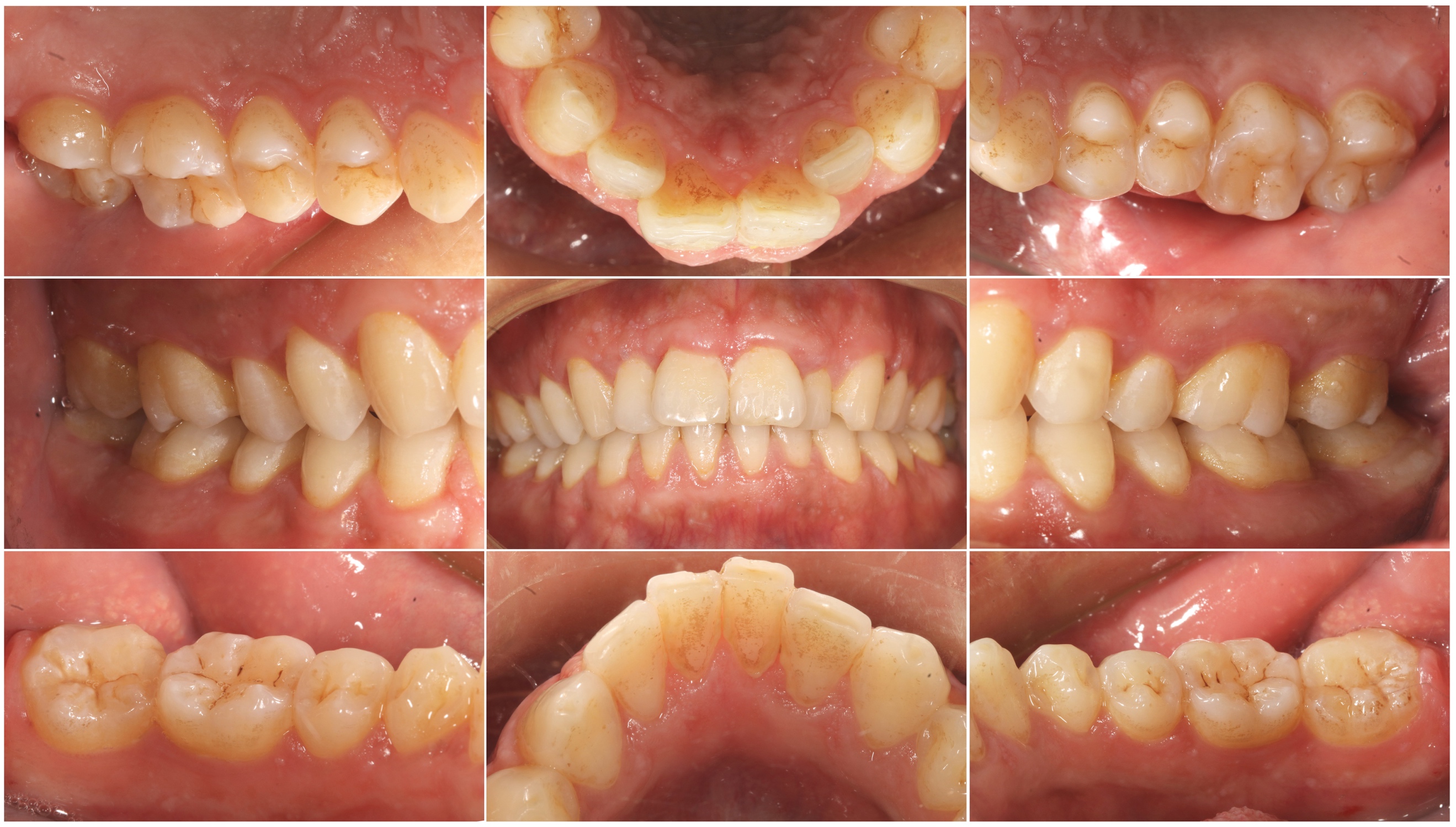

Supplement: Supplementary file 1 — Additional file 1. [file 12903_2023_3282_MOESM1_ESM.docx]
